# Unconstrained face mask and face-hand interaction datasets: building a computer vision system to help prevent the transmission of COVID-19

**DOI:** 10.1007/s11760-022-02308-x

**Published:** 2022-07-22

**Authors:** Fevziye Irem Eyiokur, Hazım Kemal Ekenel, Alexander Waibel

**Affiliations:** 1 Institute for Anthropomatics and Robotics, Karlsruhe Institute of Technology, Karlsruhe, Germany; 2 Department of Computer Engineering, Istanbul Technical University, Istanbul, Turkey

**Keywords:** COVID-19, Face mask detection, Face-hand interaction detection, Social distance measurement, CNN

## Abstract

Health organizations advise social distancing, wearing face mask, and avoiding touching face to prevent the spread of coronavirus. Based on these protective measures, we developed a computer vision system to help prevent the transmission of COVID-19. Specifically, the developed system performs face mask detection, face-hand interaction detection, and measures social distance. To train and evaluate the developed system, we collected and annotated images that represent face mask usage and face-hand interaction in the real world. Besides assessing the performance of the developed system on our own datasets, we also tested it on existing datasets in the literature without performing any adaptation on them. In addition, we proposed a module to track social distance between people. Experimental results indicate that our datasets represent the real-world’s diversity well. The proposed system achieved very high performance and generalization capacity for face mask usage detection, face-hand interaction detection, and measuring social distance in a real-world scenario on unseen data. The datasets are available at https://github.com/iremeyiokur/COVID-19-Preventions-Control-System.

## Introduction

The COVID-19 pandemic has affected the whole world since the beginning of 2020. In order to decrease the transmission of the COVID-19 disease, many health institutions, particularly the World Health Organization (WHO), have recommended serious constraints and preventions [1]. The essential precautions that individuals can carry out are practicing social distance [2], wearing a face mask properly (covering mouth and nose), paying attention to personal hygiene, especially hand hygiene, and avoiding touching faces with hands without cleanliness [1].

Convolutional Neural Networks (CNNs), introduced in late 80s [3, 4], have gained popularity during the last decade. Due to the success of deep learning in computer vision, novel research topics that emerged as a consequence of the COVID-19 pandemic are handled in this context by researchers. These studies focus on diagnosing COVID-19 [5–8], adjusting the existing surveillance systems to COVID-19 conditions [9–15], and building systems to control the preventions [10, 16–28]. Face detection and recognition systems’ performance deteriorates when subjects wear face masks. Thus, novel face recognition and detection studies [9, 11, 12, 14, 15] try to improve the performance under this condition. Moreover, in order to track the execution of preventions against the spread of COVID-19, several works investigate the detection of face masks and wearing a mask suitably [10, 16–24], how people keep physical distancing [22, 25–28], and detection of face-hand interaction [29].

To research the effects of COVID-19 regulations, some face mask datasets are introduced. In [10], a novel masked face recognition dataset is published to improve the face recognition performance in the case of occlusion due to the face masks. In [16], an artificial masked face dataset, named MaskedFace-Net, is presented. It contains 137,016 images that are generated from the FFHQ dataset [30] using a mask-to-face deformable model. Joshi et. al [17] proposed a framework to detect whether people are wearing a mask or not in public areas. They utilized MTCNN [31] and MobileNetV2 [32] to detect faces and classify them on their own video dataset. In [9], a one-stage detector based on RetinaFace [33] is proposed to detect faces and classify them whether they contain masks. In [18], the authors proposed a real-time face mask detector named SSDMNV2, which is composed of SSD [34] face detector and MobileNetV2 [32] mask classifier. In addition to the face mask detection studies, a recent study [29] investigated the face-hand touching behavior. The authors presented 2M non-touching and 74K touching face-hand interaction annotations on 64 video recordings and they evaluated introduced dataset with rule-based, hand-crafted and CNN feature-based models. As a result of evaluations, CNN-based model obtained the best results with 83.76% F1-score.

These aforementioned studies show that the face mask detection task is mostly handled in two classes, which are faces with or without a mask. However, this is not sufficient, since this setting omits improper face mask usage that frequently occurs in real-world cases. In [19], although improper usage of face mask was presented, these images are considered as no mask class when the detection system was developed. Furthermore, in [35], improper face mask class contains a small amount of images, and in [16], the images are artificially generated. In contrast to existing studies, we present a novel dataset which contains a larger set of unconstrained real world images. We handle the face mask detection as a multi-class classification task by representing improper face mask usage class as well. Differently from previous studies, we additionally aim to address face-hand interaction detection in order to prevent the spread of airborne viruses. The face-hand interaction task is investigated in [29] for the first time; however, the utilized dataset is not collected for this purpose and does not correspond to the real-world conditions. This motivates us to collect and annotate an unconstrained dataset for face-hand interaction detection as well. Since our objective is to monitor three main COVID-19 protective measures, namely face mask detection, face-hand interaction detection, and social distance measurement tasks, we develop a comprehensive computer vision system that handles these measures jointly for the first time. Moreover, we show the positive effect of using large-scale datasets of diverse facial images on the tasks’ performances and generalization capacity of the trained models.

In this work, we collected two novel face datasets, namely Interactive Systems Labs Unconstrained Face Mask Dataset (ISL-UFMD) and Interactive Systems Labs Unconstrained Face-Hand Interaction Dataset (ISL-UFHD). These datasets are collected from the web to provide a significant amount of variation in terms of pose, illumination, resolution, environment, and subjects’ ethnicities. We utilized proposed datasets for the training of presented system which consists of three submodules, face mask detection, face-hand interaction detection, and social distance measurement tasks, respectively. We trained well-known CNN models for the face mask and face-hand interaction detection tasks. While the first model classifies the face image as wearing a mask properly, wearing a mask improperly, or not wearing a mask, the second model classifies face images as touching the face or not. The trained models are evaluated both on the collected datasets and on the existing face mask datasets in the literature without training or fine-tuning on them. We also proposed a rule-based approach to measure the social distance.

Our contributions can be summarized as follows: (1) We present two novel datasets, ISL-UFMD and ISL-UFHD, for face mask and face-hand interaction detection tasks. ISL-UFMD is one of the largest face mask datasets that includes real-world images with a significant amount of variations and improper face mask usage class. The ISL-UFHD is the first dataset that contains face-hand interaction images from unconstrained real-world scenes. (2) To help people to follow protective measures to avoid spread of COVID-19, we develop a computer vision system that contains all three tasks for the first time. (3) We extensively investigate several CNN models on our datasets to show the efficiency of our unconstrained datasets. We also tested them on publicly available masked face datasets without performing adaptation, e.g. fine-tuning, on them to demonstrate the generalization capacity of our trained models. We achieved very high classification accuracies which indicates the collected datasets’ capability to represent real-world cases. Moreover, to evaluate the overall system, we utilized six different short real-world videos.

**Table 1:** Comparison of the face mask datasets

Dataset name	No mask	Mask	Improper Mask	Face Mask Type	Ethnicities	Head Pose
ISL-UFMD	10698	10618	500	Real	Various	Various
RMFD [10]*	90468	2203	–	Real	Asian	Frontal to Profile
RWMFD [10]	858	4075	238	Real	Mostly Asian	Frontal to Profile
Face mask [35]	718	3239	123	Real	Mostly Asian	Various
MaskedFace-Net [16]	–	67049	66734	Artificial	Various	Mostly Frontal

(*) Although it is stated that RMFD dataset [10] contains 5000 face images with mask, there are only 2203 face images with mask in the publicly available version

## The ISL-UFMD & ISL-UFHD datasets

Existing datasets, which are listed in Table 1, mainly focused on collecting face mask images to develop a system that examines whether there is a mask on the face. Most of them contain a limited amount of improper face mask images or include artificially generated masks on the face images using landmark points around the mouth and nose. Besides, the variety of subjects’ ethnicity, environment, resolution, and head-poses are limited. For instance, in these datasets except MaskedFace-Net [16], Asian people are in the majority. Although MaskedFace-Net includes variation in terms of ethnicity, it consists artificially generated face mask images. Besides, they have limited head-poses mostly from frontal to profile view in yaw axis. Thus, these limitations led us to collect an unconstrained dataset. Additionally, there is only one dataset [29] with face-hand interaction annotations. However, these annotations are limited based on the number of subjects and the dataset is collected under controlled conditions. In contrast, we present a face-hand interaction dataset that is collected from unconstrained real-world scenes.

### Data collection

We collected a large amount of face images from several different resources, such as publicly available face datasets, FFHQ [30], CelebA [36], LFW [37], Wider-Face [38], YouTube videos, and web. These different sources enable us to collect a significant variety of face images in terms of ethnicity, age, and gender. In addition to the subject diversity, we obtained images from indoor and outdoor environments, under different light conditions and resolutions. We also considered ensuring large head pose variations. Moreover, another important key point is to leverage the performance of our COVID-19 prevention system for the combined scenario, e.g., determining mask usage in the case of touching faces or detecting face-hand interaction in the case of wearing a mask. Besides, our images include different sorts of occlusion that make the dataset more challenging. In the end, ISL-UFMD contains 21,816 face images for the face mask detection scenario, 10,618 face images with masks and 10,698 images without a mask. Additionally, we gathered 500 images for improper mask usage. This class has a relatively small number of images compared to no mask and mask classes due to lack of face images with improper mask usage.

The ISL-UFHD is composed of 20,038 samples with and 10,018 samples without face-hand interaction. Please note that, even if the hand is around the face without touching it, we annotated images as a no interaction. Therefore, the model should be able to distinguish whether the hand is touching or being close to the face.

### Data annotation

**Fig. 1:**
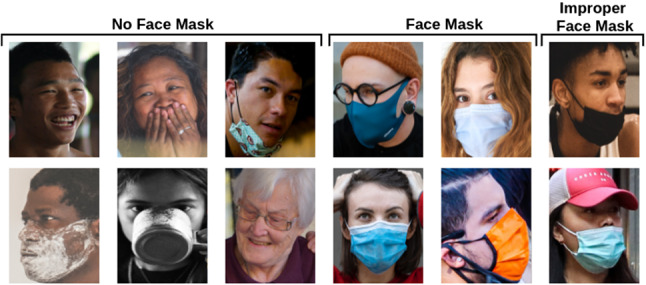
Example images from ISL-UFMD belonging to three different classes; no mask, face mask, improper face mask

**Fig. 2:**
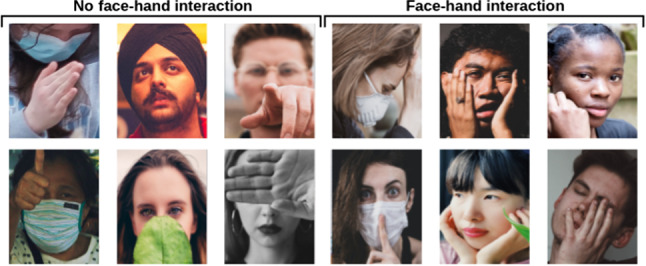
Example images from ISL-UFHD that represent face-hand interaction and no interaction

For labelling the collected datasets, we designed a web-based image annotation tool. Eleven people from different countries annotated our images using our web tool. After examining annotations from labelers, we decided each image’s final label. Since we formulate our tasks as classification problems, we annotated our images in that manner. While we have three classes—mask, no mask, improper mask—for the mask detection task, we have two classes for the face-hand interaction detection task. The images that include the face without a fully covered nose and mouth by the mask are annotated with the improper mask label. If a person has a mask under the chin, we annotated the image with no mask label. In the face-hand annotation, we aim to identify whether the hand touches the face from RGB images. We considered the direct contact or too close to contact as the existence of face-hand interaction. Many examples of annotated face images for face mask and face-hand interaction detection are shown in Figs. 1 and 2. It is clear that our proposed datasets contain large amount of variations especially for ethnicity and head pose. Also, the examples have diversity in terms of position of hand upon face and usage of face mask.

**Fig. 3:**
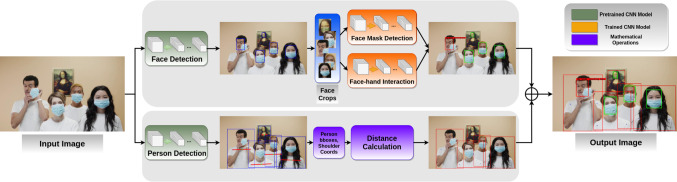
Proposed system for controlling COVID-19 preventions

## Methodology

The proposed system, which is illustrated in Fig. 3, consists of three submodules. The system performs person detection and then calculates distances between detected people on input image/video frame. Meanwhile, the same input is used to detect and crop faces of subjects to perform face mask and face-hand interaction detections. While the face mask model decides whether a person wears a mask properly, the face-hand interaction model identifies whether a hand touches the subject’s face. We decided to perform person and face detection separately to eliminate the effect of missing modality. For instance, although a person’s body is occluded and, therefore, social distancing cannot be measured for this person, system can still detect the face of the person to perform other tasks. Similarly, if the subject’s face is occluded or not turned to the camera, system can capture the person’s body to calculate the social distance.

### Face mask and face-hand interaction detection

In order to obtain face crops, we performed face detection using RetinaFace [33] that was trained on Wider-Face dataset [38]. We used RetinaFace detector since it is robust against tiny faces, challenging head poses, and faces with a mask. Then, we cropped detected faces with a 20% margin for each side, since the face detector’s outputs are quite tight. To perform face mask and face-hand interaction detections, we employed several different CNN architectures, namely ResNet50 [39], Inception-v3 [40], MobileNetV2 [32], and EfficientNet [41]. We decided to use EfficientNet, since it is the state-of-the-art model. We also included MobileNetV2, since it is a light-weight deep CNN model. Finally, we chose ResNet and Inception-v3 models based on their high performances. In the training, we benefited from transfer learning and initialized our networks with the weights of the pretrained models on ImageNet [42]. We employed softmax loss at the end of each network. In EfficientNet and MobileNetV2, we utilized dropout with a 0.2 probability rate to avoid overfitting. For training, we used 0.0001 learning rate and 0.0005 weight decay parameters. We optimized our models with Adam [43] with $\beta _1, \beta _2 = (0.9,0.999)$. The input sizes of the networks are $224\times 224$, $256\times 256$, $299\times 299$ for MobileNetV2, ResNet50, and Inception-v3, respectively. For the EfficientNet, we employed networks with input sizes between $224 \times 224$ and $300\times 300$. We executed training of our models with mini-batch size of 32 to 128 on the NVIDIA Titan RTX GPU.

**Table 2:** Face mask detection results on proposed ISL-UFMD dataset for three classes

Model	Accuracy	Precision			Recall		
		No Mask	Mask	Improper Mask	No Mask	Mask	Improper Mask
Inception-v3	**98.20%**	0.985	0.986	0.833	0.988	0.984	0.800
ResNet50	95.63%	0.965	0.954	0.636	0.973	0.973	0.389
MobileNetV2	97.91%	0.988	0.975	0.842	0.983	**0.992**	0.640
EfficientNet-b0	97.82%	0.973	0.984	**0.929**	**0.992**	0.986	0.520
EfficientNet-b1	97.91%	0.979	0.986	0.800	0.990	0.984	0.711
EfficientNet-b2	97.91%	**0.990**	0.977	0.792	0.977	**0.992**	0.760
EfficientNet-b3	98.19%	0.988	**0.990**	0.733	0.986	0.982	**0.880**

Bold values indicate the best scores

### Social distance controlling

Keeping the social distance from others is another crucial measurement to avoid spreading of COVID-19. For this, firstly, we detect each person on the image using a pretrained person detection model, DeepHRNet [44]. Thus, we obtain bounding boxes around the people and estimated pose information of each person. Principally, we focus on the shoulders’ coordinates to measure the approximate body width of a person on the image. In many studies, measurements are calculated based on the bounding box around the person. However, when the angle of the body joints and pose are considered, changes on the bounding boxes may reduce the precision of the measurements. To prevent this, we propose to use shoulders’ coordinates to measure the width and identify the middle point of shoulders line as center of the body. After performing detection and pose estimation, we generated pairs $P(p_i, p_j)$ using the combination of each detected persons. $p_i$ and $p_j$ are represent each detected person. Then, we calculated the Euclidean distance between the shoulder centers of each pair of persons. In order to decide whether these persons keep social distance between each other, we adaptively calculate a threshold for each pair individually based on the average of their body width. Since the represented measurement of the real world, expressed by pixels in the image domain, constantly changes as depth increases, we overcome this by calculating the average of the body widths of two people. Since the average shoulder width of an adult is around 40-50 cm in the real world and the required social distance between two persons is 1.5-2.0 meters, we empirically decide to select $\lambda$ coefficient as three when calculating the threshold for social distance in the pixel domain as in Eq. 1.

1 $$\begin{aligned} T_{p_i, p_j} = \lambda \times (||p_{i_{s_1}} - p_{i_{s_2}}||_2 + ||p_{j_{s_1}} - p_{j_{s_2}}||_2)/2 \end{aligned}$$

Finally, if the Euclidean distance between two persons is lower than the calculated threshold, we decide that these people do not keep sufficient social distance.

## Experimental results

In the experiments, we used our proposed datasets to evaluate our system. We handled 90% of the data for training, the remaining data are reserved equally for validation and testing. However, since the ISL-UFHD dataset contains twice more data for no interaction class than interaction class, we put aside  5,000 images from no face-hand interaction class to avoid class bias in face-hand interaction detection experiments. Further, we utilized published face mask datasets in cross-dataset experiments. We used the publicly available versions^1^ of RMFD and RWMFD [10]. RMFD includes around 2,203 masked face images. For RWMFD, we executed RetinaFace and obtained 5,171 face images from 4343 images. We used MaskedFace-Net dataset [16] which contains  130,000 face images belongs to correctly worn face masks (CMFD) and incorrectly worn face masks (IMFD) subsets. Face mask dataset (Kaggle) [35] contains 853 images. We used provided annotations to acquire 4,080 crop faces.

**Fig. 4:**
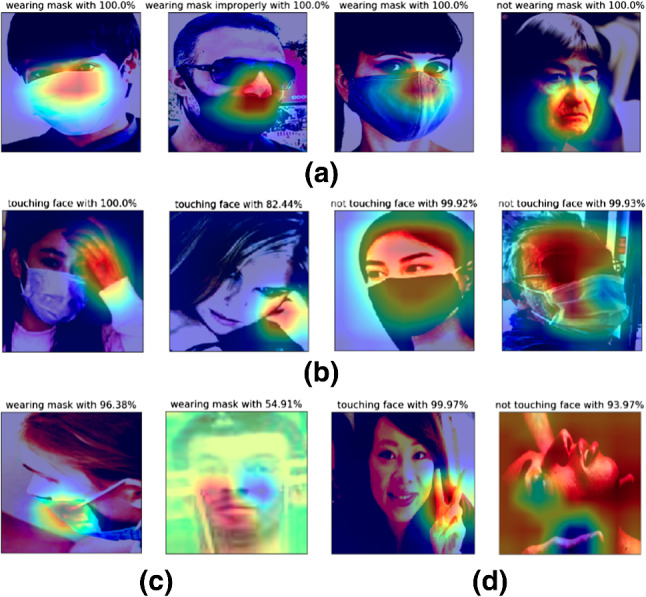
Class activation map (CAM): **a** face mask detection task, **b** face-hand interaction detection task, **c** misclassified samples of face mask detection task, **d** misclassified samples of face-hand interaction detection task

**Table 3:** Results for cross-dataset experiments. All models are trained and tested on corresponding dataset. Please note that all experiments are conducted on the 3-class classification setup to perform fair comparison

Architecture	Training Set	Test Set	# Images		Accuracy
			Train	Test	
MobileNetV2	ISL-UFMD	RMFD [10]	20764	92671	91.4%
MobileNetV2	ISL-UFMD	RWMFD [10]	20764	5171	94.7%
MobileNetV2	ISL-UFMD	MaskedFace-Net [16]	20764	133782	88.11%
MobileNetV2	ISL-UFMD	Face mask [35]	20764	4080	**95.71%**
Inception-v3	ISL-UFMD	RMFD [10]	20764	92671	**95.91%**
Inception-v3	ISL-UFMD	RWMFD [10]	20764	5171	**95.9%**
Inception-v3	ISL-UFMD	MaskedFace-Net [16]	20764	133782	**91.42%**
Inception-v3	ISL-UFMD	Face mask [35]	20764	4080	94.7%
MobileNetV2	RMFD + RWMFD	ISL-UFMD	97842	21816	86.59%
MobileNetV2	RMFD + RWMFD	Face mask [35]	97842	4080	91.07%
MobileNetV2	MaskedFace-Net + FFHQ	ISL-UFMD	211936	21816	51.49%
MobileNetV2	MaskedFace-Net + FFHQ	Face mask [35]	211936	4080	20.4%
Inception-v3	RMFD + RWMFD	ISL-UFMD	97842	21816	88.92%
Inception-v3	RMFD + RWMFD	Face mask [35]	97842	4080	88.4%
Inception-v3	MaskedFace-Net + FFHQ	ISL-UFMD	211936	21816	51.39%
Inception-v3	MaskedFace-Net + FFHQ	Face mask [35]	211936	4080	19.2%

Bold values indicate the best scores

### Face mask detection

In Table 2, we presented the results of the trainings on ISL-UFMD. According to the experimental results, although all employed models achieved significantly high performance, the best one is Inception-v3 model with 98.20% classification accuracy. In addition to the classification accuracy, we also presented precision and recall measurements for each class separately. It is also observed that the precision and recall values are very accurate for no mask and mask classes, while the results for improper mask class are slightly lower than these two classes. Even though improper face mask images may confuse with proper face mask images due to visual similarity, the more probable reason behind this outcome is the lack of images for improper mask class.

In Fig. 4a, we demonstrated Class Activation Maps (CAM) [45] for the face mask detection task to investigate activation of the model. It is clearly seen that the model focuses on the middle part of the faces, particularly on the nose and mouth. In the second image, the model identified improper mask usage since the nose of the subject is not covered by the face mask even though the mouth is covered. In Fig. 4c, we presented some misclassified images. Although the model classifies the images incorrectly, the prediction probabilities of the model are not as high as in correct predictions. This outcome indicates that the model did not confidently misclassify images. Still, the difficulty in the head pose and illumination causes misclassification in some cases.

**Table 4:** Face-hand interaction detection results on proposed ISL-UFHD dataset

Model	Accuracy	Precision	Recall
Inception-v3	93.20%	0.932	0.932
ResNet50	91.76%	0.918	0.918
MobileNetV2	92.37%	0.924	0.924
EfficientNet-b0	92.37%	0.926	0.924
EfficientNet-b1	92.90%	0.929	0.929
EfficientNet-b2	**93.35%**	**0.933**	**0.934**
EfficientNet-b3	92.44%	0.925	0.924

Bold values indicate the best scores

*Cross-dataset experiments* In the first experiment, we evaluated MobileNetV2 and Inception-v3 models, that were trained on our proposed dataset, on four different public face mask datasets. These results are presented in the first part of Table  3. We employed two different architectures to endorse experimental outcome. In the second experiment, we finetuned the MobileNetV2 and Inception-v3 models with two training setups to compare with the models that were trained on our dataset and these results are shown in the second part of Table 3. The first setup contains 97,842 images from the combination of RMFD and RWMFD datasets [10]. We used them together since RMFD dataset has no improper mask class. The second setup includes 211,936 images from the MaskedFace-Net dataset [16] with FFHQ dataset [30] due to absence of no mask class on MaskedFace-Net. While we selected RMFD, RWMFD, MaskedFace-Net, and Face mask (Kaggle) [35] datasets as target for our model, we used the proposed ISL-UFMD dataset and Face mask (Kaggle) dataset as target datasets for other models. Almost all models, that were trained on the ISL-UFMD, achieved more than 90% accuracy. These results indicate that our ISL-UFMD dataset is significantly representative to provide well generalized models for the face mask detection task. The combination of RMFD and RWMFD also provided accurate results, although they are not as high as the ones obtained by training the models on the proposed dataset. The models, that are trained on the MaskedFace-Net, show the worst performance. A possible reason of this outcome could be due to the fact that the artificial data are not as useful as the real data for the training.

### Face-hand interaction detection

In Table 4, we present the face-hand interaction detection results. As in the face mask detection task, all of the employed models have achieved very high performance to discriminate whether there is an interaction with hand. The best classification accuracy is obtained as 93.35% using EfficientNet-b2 model. The best recall and precision results are achieved by EfficientNet-b2 model as well. Almost all results in the table are considerably similar to each other. Precision and recall metrics are balanced and compatible with the accuracies.

In Fig. 4b, we provide CAM [45] for the face-hand interaction detection. It is clearly seen that the model focuses on the hand region to decide whether there is an interaction, if hand exists. In Fig. 4d, we demonstrate some misclassified images for the face-hand interaction detection. In the first image, although the model can detect the hand and the face, it cannot identify the depth between them due to the position of the hand. In the second image, the interaction with hands is not correctly classified due to the challenging angles of the head and hands.

### Social distance controlling

We utilized six different videos that we collected from the web to evaluate proposed social distancing module. These videos have different number of frames and they were recorded in various environments with different camera angles. During the calculation of the accuracy of the social distance measurement algorithm, we utilized the annotations that we decided based on the subject pairs and existing distance between each other. Person detector could not detect some of the subjects in the scene, if they are not visible in the camera due to the occlusion by other people or objects. For that reason, we ignored the missing detections when we annotated the videos’ frames and calculated the accuracies. According to the results in Table 5, we achieved very high accuracies on average. However, the fundamental problem, especially occurred in the last video, is caused by the lack of depth information. We project real-world distances to the image pixels with a rule-based approach without using reference points. Therefore, depth perception can be problematic for specific angles.

**Table 5:** Evaluation of the overall system on the test videos

Video	# frames	# sub.	Mask acc.	Face-hand acc.	Dist. acc.
V1	179	2	100%	99.16%	98.32%
V2	307	2	99.51%	96.25%	100%
V3	303	3	96.91%	89.43%	96.69%
V4	192	3	100%	86.97%	97.22%
V5	207	5	99.03%	95.45%	100%
V6	105	7	87.07%	99.86%	74.55%
Total	1293	22	97.95%	93.84%	96.51%

### Overall system performance

We evaluated the overall system performance on the same six videos and presented the results in Table 5. When we examined the face-hand interaction and face mask detection performance of our system, the results on videos that contains various people and cases indicate that system can reach very high performance similar to the ones that are obtained by the models on individual test sets.

## Conclusion

In this paper, we collected and presented unconstrained face mask (ISL-UFMD) and face-hand interaction (ISL-UFHD) datasets to conduct face mask and face-hand interaction detection tasks. Further, we proposed a system to track essential COVID-19 preventions, which are proper face mask usage, avoiding face-hand interaction, and keeping social distance, together for the first time. We employed several different well-known CNN models to perform our system and create benchmark results for our proposed datasets. Additionally, we performed geometric calculation to check the social distance between people. Experimental results showed that trained models achieved significantly high performance with the help of our proposed datasets, since they contain a large amount of variation which represents various cases in the real world. The cross-dataset experiments indicate the generalization capacity of trained models on unseen data. The proposed system can be effectively utilized to track all preventions against the transmission of COVID-19. As a future work, we will focus on to collect more improper face mask usage images to improve the performance as well as contribute to the literature by providing more data.

## Footnotes

1 https://github.com/X-zhangyang/Real-World-Masked-Face-Dataset.
